# First use of antineoplastic agents in women with breast cancer in the state of Rio de Janeiro, Brazil

**DOI:** 10.3389/fphar.2023.1069505

**Published:** 2023-02-06

**Authors:** Ranailla Lima Bandeira dos Santos, Claudia Garcia Serpa Osorio-de-Castro, Mario Jorge Sobreira-da-Silva, Vera Lúcia Edais Pepe

**Affiliations:** ^1^ Sergio Arouca National School of Public Health/Oswaldo Cruz Foundation, Rio de Janeiro, Brazil; ^2^ Department of Pharmaceutical Policies and Pharmaceutical Services, Sergio Arouca National School of Public Health/Oswaldo Cruz Foundation, Rio de Janeiro, Rio de Janeiro, Brazil; ^3^ National Cancer Institute, Rio de Janeiro, Rio de Janeiro, Brazil; ^4^ Department of Health Planning and Administration, Sergio Arouca National School of Public Health/Oswaldo Cruz Foundation, Rio de Janeiro, Rio de Janeiro, Brazil

**Keywords:** neoplasms of the breast, antineoplastic agents, medicine utilization, pharmaceutical services, unified health system

## Abstract

**Context:** Breast cancer is the most common cancer, except for non-melanoma skin cancer, among women in Brazil and worldwide. Breast cancer treatment involves surgery, radiotherapy and chemotherapy, which is used in 70% of patients. This study analyzes the utilization of antineoplastic agents among women undergoing their first round of chemotherapy in Brazil’s public health system (SUS) in the state of Rio de Janeiro.

**Methods:** Data from the SUS Outpatient Information System’s authorizations for high-complexity outpatient procedures (APACs) billed between January 2013 and December 2019 were extracted, and three datasets were created: all type 1 and type 2 APACs (including all chemotherapy procedures performed); all type 1 APACs; and first type 1 APACs (containing data only for the first round of breast cancer chemotherapy). Names of antineoplastic agents were standardized to enable the subsequent classification of therapy regimens, mitigating limitations related to data quality. Absolute and relative frequencies were used to describe sociodemographic, clinical and treatment characteristics, therapy regimen and supportive drugs.

**Results:** We analyzed 23,232 records of women undergoing their first round of chemotherapy. There was a progressive increase in the number of procedures over time. Women were predominantly white, lived in the capital and close to the treatment center. Most had stage 3 cancer at diagnosis (50.51%) and a significant proportion had regional lymph node invasion (37.9%). The most commonly used chemotherapy regimens were TAC (docetaxel, doxorubicine, cyclophosphamide) (21.05%) and and cyclophosphamide (17.71%), followed by tamoxifen (15.65%) and anastrozole (12.94%). Supportive drugs were prescribed to 386 women and zoledronic acid was predominant (59.58%).

**Conclusion:** The findings point to important bottlenecks and possible inequities in access to treatment and medicine utilization for breast cancer patients in Brazil. Efforts to improve breast cancer treatment and prevention should not only focus on interventions at the individual level but address the disease as a public health problem. The study focused on women undergoing their first round of treatment, providing valuable insight into patient and treatment characteristics to inform policy decisions.

## Introduction

Breast cancer is a complex heterogenous disease consisting of several clinical, morphological, and biological subtypes. Breast neoplasms with similar histological features and clinical presentations may have different prognoses and responses to therapy (Weigelt and Reis-Filho, 2009). The characterization of different types of breast cancer is important because it can contribute to the assessment of prognosis and cancer management. Breast cancer is a public health problem being the most common cancer, except for non-melanoma skin cancer, and one of the leading causes of death among women (Brazil. National Cancer Institute (Instituto Nacional de Câncer – INCA), 2019; Sharma, 2021; Sung et al., 2021).

In Brazil, according to estimates from the National Cancer Institute (INCA) there were 66,280 new cases of breast cancer each year during the period 2020–2022. Breast cancer has become the most common cancer among Brazilian women, accounting for 29.7% of female cancer cases. The state of Rio de Janeiro has the highest crude breast cancer incidence rate per 100,000 population in the country’s Southeast region (104.69 cases per 100,000 population) (Brazil. National Cancer Institute (Instituto Nacional de Câncer – INCA), 2019).

There are various treatment options for breast cancer, with survival rates increasing thanks to technological advances in diagnosis and treatment (Burguin et al., 2021). According to Brazil’s Breast Cancer Diagnosis and Treatment Guidelines, breast cancer treatment should be undertaken in multiple stages involving different treatment modalities, such as surgery and radiotherapy, for locoregional treatment, and chemotherapy (including hormone therapy, targeted therapy and immunotherapy) for systemic therapy (Brazil. Ministry of Health, 2018). Currently there are no published studies describing the profile of systemic therapy for breast cancer in the country.

The aim of this study was to analyze the utilization of antineoplastic agents among women undergoing their first round of chemotherapy for breast cancer on the country’s public health system, the *Sistema Único de Saúde* (SUS) or Unified Health System, in the state of Rio de Janeiro, Brazil.

## Methods

### Study design

We conducted an *exploratory cross*-*sectional* study using secondary data.

### Data extraction

Chemotherapy treatment on the SUS requires an “Authorization for High-Complexity Outpatient Procedures” (APAC, acronym in Portuguese), which contains information about the patient, indicated treatment and responsible health professional.

Data from APACs for chemotherapy were extracted from the Outpatient Information System (SIA/SUS) accessed *via* the website of the SUS’s Department of Informatics (http://www2.datasus.gov.br/DATASUS/index.php?area=0901). We collected data from APACs billed in the state of Rio de Janeiro between January 2013 and December 2019. APAC data files are publicly accessible.

### Selection of chemotherapy procedure records

APACs provide authorization for specific procedures or rounds of chemotherapy. There are two types of APAC: first cycle or type 1, which provide authorization for rounds of treatment in the first month of treatment; and continuity or type 2, which provide authorization for rounds of treatment in subsequent months. Authorization for chemotherapy is valid for 3 months after APAC approval.

The selected records were grouped into three datasets: 1) the first one included all chemotherapy procedures performed in the state during the study period (n = 422,025 records) containing APACs types 1 and 2; 2) the second included only type 1 APACs. In this dataset the same patient may appear more than once, depending on the number of rounds of treatment (n = 153,344 records); and 3) the third included type 1 APACs that specifically corresponded to the first round of chemotherapy treatment (n = 23,232 records). This third dataset was analyzed in this study.

### Study variables

The descriptive statistics were grouped into the following categories: sociodemographic characteristics; clinical characteristics and disease pathologies; and treatment characteristics. The sociodemographic characteristics included *race/color* (white; brown; black; yellow, indigenous; information not available); *age group* (under 40; 40–59; 60–79; 80 and over); *municipality of residence* (Rio de Janeiro or other); and *location of the health facility same as the municipality of residence* (yes or no). Clinical characteristics and disease pathologies consisted of *ICD-10 codes* (C50.0; C50.1, C50.2; C50.3; C50.4; C50.5; C50.6; C50.8; C50.9); *year in which the pathology was identified* (2013; 2014; 2015; 2016; 2017; 2018; 2019); *regional lymph node invasion* (invaded; not invaded; invasion undetected); and *staging* (0; 1; 2; 3; 4). Treatment characteristics included *records of systemic therapy for breast cancer* (2013; 2014; 2015; 2016; 2017; 2018; 2019); *type of systemic therapy* (chemotherapy or hormone therapy); and *classification of systemic therapy* (adjuvant; palliative; neoadjuvant).

Medicine utilization was assessed using the variables *therapy regimen* and *supportive drugs* in the treatment plan field of the APAC.

### Data analysis

The description of the profile of the utilization of antineoplastic agents is based on the analysis of the records of women undergoing their first round of chemotherapy for breast cancer during the study period.

With regard to *therapy regimen*, different names and spellings were used for the same antineoplastic agent in the APACs, making it absolutely necessary to standardize notation of antineoplastic agents (drug names, doses, and regimens) present in the administrative database. The antineoplastic agent with most differences in notation was tamoxifen, which received 350 different names and spellings. Notation was standardized independently by two researchers and any differences in opinion were resolved by a third researcher specialist in oncology.

Systemic therapy was classified into the following groups: hormone therapy; chemotherapy; hormone therapy + chemotherapy; NA (not applicable)—cases where only supportive drug(s) were administered.

Absolute (n) and relative (%) frequencies were used to describe the following variables: sociodemographic characteristics, clinical characteristics and disease pathologies, treatment characteristics, therapy regimen*,* and supportive drugs. Data cleaning and analysis was performed using R^®^ version 3.1.17 and Microsoft Excel^®^ version 2,205.

## Results

The sample consisted of 23,232 women undergoing their first round of chemotherapy for breast cancer.

The women were predominantly white (41.38%) and aged 40–59 years (46.57%). A large proportion of the women lived in the state capital (40.12%) and in 41.75% of cases the municipality of residence was different to the city where the health facility was located (Table 1).

**TABLE 1:** Distribution of women undergoing their first round of chemotherapy for breast cancer by sociodemographic, treatment, and clinical characteristics and disease pathology. State of Rio de Janeiro, Brazil (2013–2019).

	n	%
SOCIODEMOGRAPHIC CHARACTERISTICS		
Race/color		
White	9,614	41.38
Brown	7,286	31.36
Information not available	3,347	14.41
Black	2,799	12.05
Yellow	183	0.79
Indigenous	3	0.01
Age group (years)		
40–59	10,818	46.57
60–79	9,245	39.79
Under 40	1,776	7.63
80 and over	1,393	6.00
Municipality of residence		
Rio de Janeiro	9,320	40.12
Other	13,912	59.88
Location of the health facility same as the municipality of residence		
Yes	13,532	58.25
No	9,700	41.75
CLINICAL CHARACTERISTICS AND DISEASE PATHOLOGIES		
ICD-10 CODES		
Malignant neoplasm of upper-outer quadrant of breast (C50.4)	6,646	28.61
Malignant neoplasm of breast, unspecified (C50.9)	5,910	25.44
Malignant neoplasm of nipple and areola (C50.0)	3,960	17.05
Malignant neoplasm of central portion of breast (C50.1)	3,641	15.67
Overlapping lesion of breast (C50.8)	1,284	5.53
Malignant neoplasm of lower-inner quadrant of breast (C50.3)	681	2.93
Malignant neoplasm of axillary tail of breast (C50.6)	436	1.88
Malignant neoplasm of lower-outer quadrant of breast (C50.5)	346	1.49
Malignant neoplasm of upper-inner quadrant of breast (C50.2)	328	1.41
Year in which the pathology was identified		
2013	3,963	17.06
2018	3,827	16.47
2014	3,339	14.37
2017	3,296	14.19
2015	3,280	14.12
2016	3,130	13.47
2019	2,397	10.32
Regional lymph node invasion		
Invaded	8,805	37.90
Unassessable	8,052	34.66
Not invaded	6,375	27.44
Staging		
3	11,734	50.51
2	5,534	23.82
1	3,022	13.01
4	2,479	10.67
0	463	1.99
TREATMENT CHARACTERISTICS		
Records of systemic therapy for breast cancer		
2019	4,192	18.04
2018	4,004	17.23
2017	3,366	14.49
2015	3,362	14.47
2016	3,251	13.99
2014	2,979	12.82
2013	2,078	8.84
Type of systemic therapy		
Chemotherapy	15,574	67.04
Hormone therapy	7,658	32.96
Classification of systemic therapy		
Adjuvant	11,728	50.48
Neoadjuvant	7,885	33.94
Palliative	3,619	15.58

Source: Outpatient Information System (SIA/SUS).

The number of cases diagnosed each year decreased over the study period from 3,963 in 2013 (17.06% of total cases) to 2,397 in 2019 (10.32%). The most common diagnosis was “Malignant neoplasm of upper-outer quadrant of breast” (C50.4) (28.61%).

The most common stage of cancer at the time of diagnosis was stage 3 (50.51%) for white and non-white women alike (Table 1). However, stages 0–2 showed larger proportions among white women compared non-white women (data not shown). Most of the cases had regional lymph node invasion (37.90%) (Table 1).

The results show that there was a progressive increase in the number of procedures performed over the study period. Chemotherapy was used in 67.04% of the cases. The most common type of systemic therapy was adjuvant therapy (after surgery), used in 50.48% of the women (Table 1).

Table 2 shows that 386 women (1.70%) were prescribed supportive drugs. The most commonly used drug was zoledronic acid (59.58%), followed by pamidronate (29.28%)**.**

**TABLE 2:** Absolute and relative frequencies of supportive drugs in the High Complexity Outpatient Procedure Authorization. State of Rio de Janeiro, Brazil (2013–2019).

Supportive drugs	n	%
Zoledronic acid	230	59.58
Pamidronate	113	29.28
Filgrastim	19	4.92
Osteolysis inhibitor	13	3.37
Folinic acid	7	1.81
Ondansetron	3	0.78
Dexamethasone	1	0.26
Total	386	100

Source: Outpatient Information System (SIA/SUS).

The findings show that 140 types of therapy regimens were used, comprising 111 combination therapies, 26 antineoplastic agents used alone, and three drug classes (taxanes, LHRH analogues, and aromatase inhibitors) (Table 3).

**TABLE 3:** Distribution of women undergoing chemotherapy for breast cancer by main therapy regimens in the High Complexity Outpatient Procedure Authorization. State of Rio de Janeiro, Brazil (2013–2019).

Therapy regimen	N	%
TAC	4,884	21.1
AC	4,113	17.7
Tamoxifen	3,634	15.7
Anastrozole	3,006	13.0
FAC	1,471	6.3
Paclitaxel	767	3.3
TAC + trastuzumab	509	2.2
Docetaxel	495	2.1
TC	478	2.1
AC + taxane	474	2.0
CMF	390	1.7
Carboplatin + Paclitaxel	372	1.6
AC-T	294	1.3
FEC	291	1.3
Trastuzumab	230	1.0
Exemestane	221	1.0
FAC + docetaxel	195	0.8
Capecitabine	153	0.7
Docetaxel + Trastuzumab	128	0.6
Doxorubicin	128	0.6
Cyclophosphamide	86	0.4
Cisplatin + Gemcitabine	79	0.3
AC-TH	63	0.3
TCH	60	0.3
Paclitaxel + Trastuzumab	44	0.2
Anastrozole + Tamoxifen	39	0.2
FEC + docetaxel	39	0.2
Fulvestrant	33	0.1
Tamoxifen + Trastuzumab	26	0.1
Vinorelbine	26	0.1
Others	444	1.9
Total	23,172	100

Source: Outpatient Information System (SIA/SUS).

Notes: TAC: docetaxel, doxorubicine, cyclophosphamide; AC: doxorubicin and cyclophosphamide; TCH: docetaxel, carboplatin and trastuzumab; AC-TH: doxorubicin and cyclophosphamide + paclitaxel and trastuzumab; TC: docetaxel and cyclophosphamide; AC-T: doxorubicin and cyclophosphamide + paclitaxel; FAC: cyclophosphamide, doxorubicin and 5-fluorouracil; CMF: cyclophosphamide, methotrexate and 5-fluorouracil; FEC: cyclophosphamide, epirubicin and 5-flouracil.

The most commonly used therapy regimens were the combination therapies TAC (docetaxel, doxorubicine, cyclophosphamide) (21.05%) and AC (doxorubicin and cyclophosphamide) (17.71%), followed by tamoxifen (15.65%) and anastrozole (12.94%). Together, these regimens account for 68% of all treatments (Table 3).

## Discussion

We analyzed 23,232 records of women undergoing their first round of chemotherapy. Number of procedures increased over time. Women were predominantly white, lived in the capital and close to the treatment center. Most had stage 3 cancer at diagnosis and a significant proportion had regional lymph node invasion. The most commonly used chemotherapy regimens were TAC (docetaxel, doxorubicine, cyclophosphamide) and AC (doxorubicin and cyclophosphamide), followed by tamoxifen and anastrozole. Zoledronic acid was the most prescribed supportive treatment.

Our findings provide some valuable insights into sociodemographic, clinical and treatment characteristics, and medicine utilization among women undergoing their first round of chemotherapy on the SUS in the state of Rio de Janeiro. Previous studies have investigated chemotherapy procedures as a whole without focusing specifically on women, using type 1 APACs or a combination of type 1 and 2 APACs (Atty et al., 2017; Silva et al., 2019a; Saldanha et al., 2019).

Publicly accessible databases are important tools for monitoring public policies and programs (Malta et al., 2006; Rodrigues-Júnior, 2012). APACs have broad population coverage and include data on medicines used on the SUS, providing a useful tool for monitoring medicine utilization and health programs (Soares and Silva, 2013).

The predominance of white women found by the present study corroborates the findings of other studies (Guimarães and Anjos, 2012; Medeiros et al., 2015; Rocha et al., 2020). The higher prevalence of white women in our dataset may be a reflection of delays in cancer diagnosis and poor access to care among black women (Marcelino et al., 2021). In this regard social inequalities can result in poorer health status and disparities in access to and utilization of health services (Cabral et al., 2019).

Most of the women in our sample were aged between 40 and 59 years, which is consistent with the literature reporting that the prevalence of breast cancer is lower in women age aged under 40 (Silva et al., 2021; Sopik, 2021). However, studies have shown an increase in breast cancer incidence and mortality in Brazil and worldwide across all age groups, including younger women. Evidence also demonstrates that young patients are more likely to have more aggressive clinicopathological characteristics, increased risk of recurrence, lower disease-free and overall survival, and to be diagnosed with more advanced stage cancer (Zhang et al., 2018; Orlandini et al., 2021).

A large proportion of the women (42%) lived outside the city where the health facility was located, corroborating the findings of a nationwide study by Silva et al. (2019a) showing that 49.2% of patients undergoing chemotherapy for breast cancer had to travel to another city to receive treatment. Our findings also show that patients who lived in the municipality where the health facility was located had a greater chance of accessing services, concurring with the relevant literature on this topic (Rodrigues et al., 2015; Wan and Jubelirer, 2015). Studies show that geographic access and distance to health services are critical factors determining cancer care and can lead to delay in diagnosis, meaning that cancer is often at a more advanced stage when diagnosed, consequently affecting chances of survival (Oliveira et al., 2011; Piñeros et al., 2011; Silva et al., 2019b).

Our findings show that more than half of the women in our sample, regardless of race/color, had stage 3 cancer (advanced) at diagnosis, concurring with the literature (Azevedo e Silva et al., 2004; Brito et al., 2005; Cintra et al., 2008). In a study using data from type 1 APACs, Atty et al. (2017) reported that stages 2 and 3 were the most common stages of breast cancer at diagnosis at national level and across all regions except the South, where stage 1 was the second most common stage. Discrepancies among white and non-white women were observed; however, proportions of lower cancer stages, albeit predominant among white women when compared to non-white women, were low in both groups.

In addition to the natural history of cancer, difficulties in accessing health services and poor early diagnosis capacity limit treatment alternatives and result in late-stage diagnosis and lower likelihood of a cure (Frazão and Skaba, 2013). In a study with women with breast cancer receiving treatment on the SUS in the state of Rio de Janeiro, Brito et al. (2005) reported low rates of early (stage 1) cancer diagnosis.

The data presented show that most of the cases (37.90%) were characterized as tumors with regional lymph node invasion. According to Cintra et al. (2008), the greater the degree of lymph node invasion, the lower the likelihood of survival. Kalinsky et al. (2021) highlight that the benefit of adjuvant chemotherapy is unclear in patients with positive lymph nodes.

The findings show that there was a progressive increase in the number of chemotherapy procedures, suggesting an increase in the quantity and quality of diagnoses, which in turn indicates improved access to health services (Silva et al., 2019b).

The choice of breast cancer therapy is based on a number of criteria, including tumor histology, hormone receptor expression, axillary lymph node status, HER2 status and presence of metastases, as well as patient characteristics, such as menopausal status, age and comorbidities (Waks and Winer, 2019). The most common types of systemic therapy in our sample were chemotherapy and hormone therapy. There is consensus in the literature that hormone therapy is the most commonly used treatment as ER-positive breast cancer has become the most common type of breast cancer diagnosed today (Britt et al., 2020).

The most common classification of systemic therapy was adjuvant therapy (after surgery), which accounted for 50% of cases. Adjuvant systemic therapies, including endocrine therapy, anti-HER2 therapy, and chemotherapy are effective in reducing the risk of recurrence of breast cancer (Anampa et al., 2015).

The most commonly used therapy regimens were TAC (docetaxel, doxorubicine, cyclophosphamide), AC (doxorubicin and cyclophosphamide), and tamoxifen and anastrozole. The predominance of TAC and AC in our sample, used in around 21% and 18% of the women, respectively, is consistent with the findings of Lôbo et al. (2014), who reported that TAC and AC represented 37.2% and 12.4% of the therapy regimens, respectively. It is worth highlighting that, despite advances in breast cancer treatment (Burguin et al., 2021), the main regimes used today continue to be chemotherapy and hormone therapy using tamoxifen and anastrozole. This is especially the case in low- and middle-income countries, due to the good response and tolerance of patients to treatment and to fact that these drugs are widely-used for breast cancer and low cost (Birnbaum et al., 2018).

Tamoxifen was used in 15.7% of the cases. Although this drug contributes to improved survival in patients with larger tumors (Dar et al., 2021), poor adherence to treatment with tamoxifen remains a challenge, due to the length of treatment and associated toxicities. More than 50% of patients do not fully adhere to treatment, indicating that health services need to adopt measures to encourage greater adherence in order to improve treatment effectiveness (Montagna et al., 2021).

The aromatase inhibitor anastrozole was used in 13% of the women in our sample. The literature shows that the use of aromatase inhibitors may be associated with increased risk of cardiotoxicity. However, Lund & Ejlertsen (2022) reported that improved recurrence outcomes in most patients when using aromatase inhibitors versus tamoxifen outweighed the potential risk of adverse cardiovascular events. Standard cardiovascular disease control and prevention strategies should be promoted, including changes in life style and medical treatment for the disease and risk factors.

Supportive drugs were prescribed to 1.70% of the women. The most commonly used drugs were zoledronate (in 60% of cases) and pamidronate (in 30% of cases), both of which are bisphosphonates. In bone metastases, bisphosphonates prevent or retard skeletal-related events and can improve pain control. Bisphosphonates significantly reduce distant recurrence and bone recurrence, an effect that is observed during post-menopause, and breast cancer mortality. Although bisphosphonates are well tolerated by patients, they can lead to severe adverse events, including osteonecrosis of the jaw and kidney failure, especially in patients with metastatic cancer receiving high doses of the drug (Goldvaser and Amir, 2019; Coleman, 2020; Jackson et al., 2021). It is important to highlight that it is not possible to confirm that only a small proportion of women from our sample were prescribed these drugs because the inclusion of supportive drugs in the APAC is not mandatory.

This study has some limitations. First, the APAC is a management tool used by the public health system and it is therefore important to take into consideration possible data incompleteness, inconsistencies in the completion of APACs, and inaccuracies and missing information due to data entry errors. It is essential to involve health managers in this process and provide adequate training for health professionals to ensure the accurate completion of APACs in order to improve information quality. Second, the fact that the analysis was based on type 1 APACs meant that it was not possible to investigate medicine utilization throughout the rest of the course of treatment, as treatments authorized by subsequent APACs could not be considered. A longitudinal analysis covering the full course of treatment requires record linkage, which was not possible in this study.

As the study excludes women diagnosed with breast cancer before January 2013, the results should not be generalized to the general population of women undergoing treatment for breast cancer.

## Conclusion

Our findings highlight the importance of using data produced by health services, which, despite the quality limitations of secondary data, can provide valuable insights into disease control and patient profiles, and help tackle breast cancer. Breast cancer should be recognized as a problem that has reached epidemic proportions, becoming the leading cause of cancer deaths among women in Brazil. This argument is reinforced by the current context, characterized by late diagnosis and inequity in access to treatment. Efforts to improve breast cancer treatment and prevention should not only focus on interventions at the individual level but address the disease as a public health problem where timely diagnosis and treatment are vital to ensuring favorable outcomes. This study focused on 23.232 women undergoing their first round of treatment, providing valuable insight into patient and treatment characteristics to inform policy decisions.

## Data Availability

Publicly available datasets were analyzed in this study. The data used in this analysis is available online from the Outpatient Information System (http://www2.datasus.gov.br/DATASUS/index.php?area=0901), a publicly accessible database.
